# Wild great and blue tits do not avoid chemical cues of predators when selecting cavities for roosting

**DOI:** 10.1371/journal.pone.0203269

**Published:** 2018-09-19

**Authors:** Luisa Amo, Gustavo Tomás, Irene Saavedra, Marcel E. Visser

**Affiliations:** 1 Department of Animal Ecology, Netherlands Institute of Ecology (NIOO-KNAW), Wageningen, The Netherlands; 2 Departamento de Ecología Evolutiva, Museo Nacional de Ciencias Naturales (CSIC), Madrid, Spain; 3 Departamento de Ecología Funcional y Evolutiva, Estación Experimental de Zonas Áridas (CSIC), La Cañada de San Urbano, Almería, Spain; Universita degli Studi di Milano-Bicocca, ITALY

## Abstract

Small birds use cavities for roosting to decrease the thermoregulatory costs during the winter nights. The ability of birds to detect and escape from an approaching predator is impaired during roosting and thus the selection of such cavities should take into account the risk that a predator will find the cavity. Previous evidence suggested that birds in captivity are able to detect predator scent and avoid roosting in nest-boxes containing such predator chemical cues. Here, we tested whether birds also show this avoidance response under natural conditions. We performed three studies in three populations of blue and great tits. We added predator scent, a pungency scent or an odourless control to nest-boxes and compared the use of these nest-boxes for roosting. We found no differences between the scent treatments in the use of nest-boxes. Therefore, chemical cues indicating the potential presence of a predator are not enough for birds to avoid roosting in nest-boxes under natural conditions.

## Introduction

During winter, bird mortality is reported to be higher than at other moments of the year [1,2] especially after particular bad weather events such as frosts or storms [3]. This increased mortality during winter can have several causes. It can be due to the reduction of the available day light hours for feeding. Or, for instance for insectivorous birds, the decrease in insect abundance in winter leading to increased foraging effort [4]. Another potential cause is the increased metabolic costs associated to thermoregulation under low environmental temperatures. These costs are higher for small birds because their reduced body size involves a high energy expenditure for thermoregulation [4]. Therefore, survival of small birds can be seriously compromised during winter, especially during the night, when temperature reaches its minimum.

Birds, as well as other endotherm animals, have developed behavioural and physiological adaptations to survive hard winter conditions. Birds may increase the insulating properties of the plumage [5,6], form aggregations during the night for energy saving [7], or use cavities for roosting to minimize heat loss [8,9]. The use of cavities for roosting is especially important during the winter period for many small bird species inhabiting temperate areas. This is because during at night, when temperatures drop, birds gain thermal benefits and save energy spending the night inside a cavity compared to the canopy [10–14]. Many bird species can also decrease their body temperature during cold nights in order to decrease the costs associated with the maintenance of a constant and elevated body temperature [15,16]. By entering such nocturnal hypothermia, birds reduce their metabolic rate at night, and thereby their starvation risk. However, this decrease in temperature also entails changes in the sleep pattern [17] that may cause a lower ability to detect an approaching predator while sleeping [18]. In addition, roosting in a cavity has as a disadvantage that it is hard to escape the predator when found. Therefore, it is essential for birds to select safe cavities to roost in.

When selecting safe cavities for roosting, birds may assess the risk that such a cavity can contain, or will be visited by, a predator. This risk assessment needs to be made before entering because inside the cavity a bird has low possibilities of escaping once a predator has launched an attack. Previous evidence suggested that birds avoid roosting in nest-boxes containing cues from predators such as fur or signs of predation such as bird feathers [19]. A similar avoidance response of predator fur has been found when birds select nest-boxes for breeding [20]. Besides visual cues of predator fur, chemical cues contained in the predator fur used in these two studies may be used by birds to assess the risk of predation.

The detection of predator chemical cues may provide birds a first warning of the risk that the cavity can be occupied by a predator [21]. Visual detection of predators inside cavities can be constrained due to low visibility conditions and therefore, the use of predator chemical cues for ascertaining predator presence may be crucial to avoid being predated. Therefore, many species avoid the use of cavities containing predator chemical cues [22–24]. The presence of predator chemical cues inside a cavity may indicate both that a predator is currently inside the cavity and that a predator has previously visited the cavity and may return to it.

Previous evidence suggests that predator chemical cues can be used by birds for predation risk assessment. For example, when blue tits *Cyanistes caeruleus* found predator chemical cues inside a nest-box during the breeding period, they delayed their entry to the nest-box and decreased the time spent inside it while feeding the nestlings [25]. It was also shown that blue tits did not decrease the provisioning rate to nestlings, but they decreased the time devoted to other activities not essential for nestling survival such as nest sanitation activities [26]. Other species have also been shown to avoid predator chemical cues in a foraging context, such as the house finch, *Carpodacus mexicanus* [27, 28] and the domestic fowl, *Gallus gallus domesticus* [29]. Birds can also avoid nesting in areas containing predator scent, as *Anas* sp. exposed to fox scent did [30]. Moreover, male house sparrows (*Passer domesticus*) avoided the scent of a potential predator or competitor (*Mus musculus domesticus*) when inspecting nest-boxes for roosting [31]. However, in other cases, the presence of predator scent did not affect nesting preferences (*Sialia sialis* [32]) or parental behaviour (*Troglodytes aedon* [33]); or nesting was not avoided (*Puffinus pacificus* [34]).

The presence of predator cues does not always need to lead to avoiding a location. Even when there are such cues this does not necessarily will lead to a predator attack. Thus, any anti-predatory response should be traded off against other requirements [35, 36]. In the case of predator chemical cues, such cues may reveal the presence of predators [21], even in the absence of any other cue [21, 25, 26, 37, 38]. But the use of chemical cues can lead to an overestimation of predation risk if birds continue avoiding the area containing such cues even when the predator is no longer present [21, 39]. Therefore, if the avoidance response is costly, birds may opt to not to avoid places containing predator chemical cues, especially in the absence of other cues signalling predator presence.

The results of a previous study in captivity with great tits, *Parus major*, shows that they avoid roosting in nest-boxes containing predator scent [40]. However, to our knowledge no study has examined whether birds use predator chemical cues to assess the risk of predation when selecting cavities for roosting under natural conditions.

Here, we examined whether birds avoid roosting in nest-boxes containing predator chemical cues during winter. We performed three experiments in which we added either predator scent, an odorous control or an odourless control to nest-boxes during the morning, and checked whether the nest-boxes where used for roosting. The experiments were performed in three different populations of blue tits and great tits that use the nest-boxes for roosting during the winter period. Both species are known to be able to detect and avoid predator scent [25, 26, 40]. Moreover, great tits in captivity are known to avoid nest-boxes containing predator scent for roosting [40]. Thus, we hypothesized that also wild birds will avoid roosting in nest-boxes containing predator scent.

## Methods

### Ethics note

According to the Spanish laws in relation to animal research, the study that we report here does not need to be evaluated by an animal research ethics committee, as we did not manipulate animals. Licenses to perform the study were approved by the INAGA (500201/24/2015/11696, Spain) and the Animal Experimental Committee of the KNAW (DEC protocol no CTE 07.08, the Netherlands). The study did not involve any pain to animals, so no anaesthesia or euthanasia was required.

### Study systems and experimental designs

Experiments were performed during the winter in 2006, 2008 and 2016. In the three experiments, the experimental protocol consisted in placing a paper soiled with the correspondent treatment (predator scent, odorous control and odourless control) inside the nest-boxes during the morning. The presence of previous bird faeces was recorded and faeces were removed from nest-boxes when present. The following morning, nest-boxes were checked and we determined whether a nest-box had been occupied by a bird during the night by examining the presence of faeces inside the nest-boxes, and papers were removed. This is a reliable method to assess the use of a nest-box because birds always defecate during the night [18, 19, 40]. Therefore, we could analyse whether birds avoided to roost in cavities containing a predator scent.

As predator scent we use mustelid scent in all three experiments but we used different pungent scents as odorous control (see below). The odorous control allows us to compare the behaviour of birds when they find the odour of a predator inside the nest-box or a new pungent odour without biological significance. We used water as an odourless control to resemble the level of humidity of the papers containing the two other treatments. Water has been widely employed as an odourless control stimulus in studies on chemical detection [23, 25, 26, 40].

#### Experiment 1

In February 2006, an experiment was performed in 80 nest-boxes located in a *Quercus pyrenaica* oak forest in Madrid province (Sierra de Guadarrama, Central Spain, 40° 43´N, 03° 55´W). In this area, blue tits are more abundant than great tits during the winter (14.5 birds/10 ha vs 8.6 birds/10 ha, respectively [41]). Forty eight nest-boxes were cleaned three weeks before the experiment and contained no nest. The other 32 nest-boxes were not previously cleaned because they were going to be used in different experimental studies during the next spring. Twenty two of them contained an old nest and 10 were empty. Treatments were placed at the bottom of the nest-box and under the nest when there was a nest inside the nest-box. This methodology has been previously used in studies about the olfactory capacity of birds to detect predator scent inside nest-boxes [25, 26, 40]. The treatments were: a) mustelid scent (predator chemical cues), b) cologne (Eau de Cologne from Hema^®^, pungency control), and c) water (odourless control) (see below for details).

#### Experiment 2

In January 2008, an experiment was performed in 47 nest-boxes in a *Quercus robur* oak forest in Oosterhout (Central Netherlands, 51° 55´N, 05° 50´E). In this area, 99% of roosting birds were great tits and only 1% were blue tits. Nest-boxes did not contain any nest as they were cleaned at the end of the previous breeding season. Treatments were placed inside cotton bags that were hanged from one of the walls inside the nest-box. The treatments were: mustelid scent (predator chemical cues), b) vine vinegar (pungency control), and c) water (odourless control) (see below for details).

#### Experiment 3

In March 2016, an experiment was performed in 100 nest-boxes in a *Quercus pyrenaica* forest in Zaragoza (Alto Huerva-Sierra de Herrera, Aragón, Northeast Spain, 40° 59′ N, 01° 05′ W). In this area, blue tits are more abundant than great tits, at least during the breeding period, with 67 nest-boxes occupied for breeding, 76% by blue tits and 24% by great tits. Old nests were not previously removed from nest-boxes, so most of the nest-boxes (92%) contained a nest. Treatments were placed on the bottom of the nest-box, and under the nest when there was a nest. The treatments applied to the nest-boxes were: mustelid scent (predator chemical cues), b) lemon essence (pungency control), and c) water (odourless control) (see below for details). The following morning, nest-boxes were inspected for faeces. In this experiment, faeces were removed if present, and papers were replaced by papers with another treatment. The subsequent day, after nest-box inspection, the third treatment was applied to nest-boxes. Therefore, all nest-boxes had the three treatments in three consecutive days. The order of treatments was randomized.

### Preparation of experimental treatments

Treatments were added in an absorbent paper (12 x 7 cm). To obtain predator scent, we placed clean absorbent papers inside the cages of several male ferrets (*Mustela furo* L., ≥ 2 ferrets per experiment). Ferret scent is recognized and avoided by blue tits and great tits [25, 26, 40]. Ferrets were individually housed in cages and had water and food (dry pellets for ferrets) *ad libitum*. We placed papers in the ferret cages three days before the experiment, to ensure odour collection. When collecting papers daily for the experiment, we selected wet papers containing fresh urine. This method for collection of predator scent has been used in previous studies [25, 26, 40].

We used different pungent odorous controls in the three experiments. In experiment 1 (2006), we used cologne as a pungent control. The cologne treatment was obtained by placing 2 ml of 50% diluted cologne (Eau de Cologne from Hema^®^) on clean absorbent papers. We used cologne as an odorous control, as it was previously used in studies exploring predator scent detection in birds [40]. In experiment 2 (2008), we used vinegar as a pungent control. This treatment was obtained by placing 2 ml of 50% diluted vine vinegar on absorbent papers. Vinegar is also a pungent control that may not have biological significance for birds, and it has been previously used as a pungent scent in studies about predation risk assessment via olfaction in birds [20]. In experiment 3 (2016), we used lemon essence, a pungent control that has been previously used for experimental assessment of bird ability to detect scents inside nest-boxes [42]. To prepare lemon essence, we mixed 0.5 g of grated lemon zest in 1 ml of distilled water. The mixture was maintained 24 hours in the fridge and 2 ml from the liquid fraction were placed in absorbent papers.

We prepared the odourless control treatment by adding 2 ml of water to a clean absorbent paper. Odourless control treatments have been used in previous studies about avian olfaction [25, 26, 40].

### Statistical analyses

Statistical analyses were performed with the Statistical package R 2.15.1 [43].

#### Experiment 1

A generalized linear model (GLM), with binomial errors and a logit link function, was used to analyse whether occupancy of nest-boxes differed in relation to the treatment and to the presence of a nest inside the nest-box. We included the interaction between the treatment and the presence of a nest in the model.

Because differences between nest-boxes may influence the preference of birds for roosting in nest-boxes, we considered the use of the nest-box the previous day in the analysis of nest-box occupancy. Therefore, we performed a generalized linear model to examine whether there were differences in nest-box use considering only the nest-boxes that were previously occupied (ascertained by the presence of faeces before the experiment), analysing whether there were differences in the change in use of occupied nest-boxes between treatments.

#### Experiment 2

A generalized linear model (GLM), with binomial errors and a logit link function, was used to analyse whether occupancy of nest-boxes differed in relation to the treatment. To take into account the previous use of the nest-box, we also used a generalized linear model (GLM), with binomial errors and a logit link function, to determine whether the treatment affected the use of nest-boxes, considering only those nest-boxes that were previously used before the experiment.

#### Experiment 3

A generalized linear mixed model (GLMM), with binomial errors and a logit link function, was used to analyse whether occupancy of nest-boxes was affected by the treatment and the order of treatment presentation (fixed factor with 6 levels), including nest-box as a random factor.

We also used a generalized linear mixed model to evaluate whether the treatment affected the change in use of nest-boxes, considering only those nest-boxes that were previously used (with nest-box as a random effect), including the order of treatment presentation as a fixed factor.

## Results

### Experiment 1

In 2006, the occupancy rate of nest-boxes in Central Spain was not related to the treatment (Z = -0.38, p = 0.71; Table 1). The presence of a nest inside the nest-box did not influence nest-box use (Z = 1.46, p = 0.15) although ten out of the 22 nest-boxes (45%) that contained a nest were used to spend the night whereas only 7 of 58 nest-boxes (12%) that did not contain a nest were used. The interaction between the treatment and the presence of a nest was not significant (Z = -0.28, p = -0.78). The overdispersion value was 0.95.

**Table 1:** Number of nest-boxes used for roosting/number of nest-boxes with the correspondent treatment (and the percentage of used nest-boxes) when the nest-boxes contained predator scent (mustelid), a pungent odorous control or water in three experiments aimed to analyse whether birds avoid roosting in nest-boxes containing predator chemical cues.

	Nest-boxes occupied		
Treatment	Experiment 1 (Central Spain)	Experiment 2 (The Netherlands)	Experiment 3 (Northeast Spain)*
**Predator odour**	5/26 (19%)	4/17 (24%)	22/100 (22%)
**Odorous control**	5/27 (14%)	6/15 (40%)	21/100 (21%)
**Water**	7/27 (26%)	5/15 (33%)	23/100 (23%)
**Total**	17/80 (15.5%)	15/47 (31%)	66/300 (22%)
**Treatment effect**	Z = -0.38, p = 0.71	Z = -0.62, p = 0.54	Z = -0.41, p = 0.69

*In the experiment 1 and 2, treatments were applied only once to each nest-box, whereas in the experiment 3, a repeated measures design was used, and nest-boxes contained the three treatments in three consecutive days in a randomized order.

When considering only the nest-boxes that were previously used, there were not significant differences between treatments in the number of nest-boxes that were used before the experiment and not used during the scent exposition in relation to the treatment (Z = 0.71, p = 0.48). The presence of a nest did not influence the use of these previously used nest-boxes (Z = -0-77, p = 0.44). The interaction between the treatment and the presence of a nest did not influence the occupancy of previously used nest-boxes (Z = 0.10, p = 0.92). The overdispersion value was 1.42.

### Experiment 2

The occupancy rate of nest-boxes in 2008 in the Netherlands did not differ between treatments (Z = -0.62, p = 0.54; Table 1). The overdispersion value was 1.30. There were not significant differences between treatments in the occupancy rate of nest-boxes that were previously used before the experiment (Z = 0.003, p = 0.99). The overdispersion value was 0.52.

### Experiment 3

In 2016, in the nest-box population of north-eastern Spain, there were not differences between treatments in the occupancy of nest-boxes (Z = -0.41, p = 0.69; Table 1). The order of treatment presentation did not influence its occupancy (Z = -0.43, p = 0.67). The overdispersion value was 0.60. When considering only the nest-boxes that were previously used, neither the treatment (Z = 0.36, p = 0.72) nor the order of treatment presentation (Z = 1.29, p = 0.20) influenced the occupancy of these previously used nest-boxes. The overdispersion value was 1.16.

## Discussion

Our results show that, under natural conditions, birds did not avoid roosting in nest-boxes containing predator scent. A similar number of birds roosted inside nest-boxes containing predator scent or other treatments in the three study areas. Also, when considering the previous use of the nest-boxes (i.e. whether a bird already roosted inside a particular nest-box before adding the scents to the nest-box), our results show that birds did not avoid to roost in those nest-boxes that contained predator scent. This is in contrast with the results of a previous study performed in captivity, which found that great tits did avoid roosting in nest-boxes containing predator scent [40]. These differences in results between the two experiments cannot be attributed to the species tested because the species that roost in nest-boxes in the three areas are mainly blue tits and great tits, and both are known to detect and avoid predator scent [25, 26, 40]. Furthermore, at least in great tits, the detection of a predator scent seems to be innate, because adult birds naïve to predators avoided roosting in nest-boxes containing predator scent [40]. What is more, the protocol to collect predator scent as well as the methodology to locate scented papers inside the nest-boxes have been successfully used for measuring the capability of birds to detect predator scent [25, 26, 40]. Therefore, this previous evidence suggests that the lack of an avoidance response of birds cannot be explained by a lack of detection of the scent due to methodological problems.

In contrast, during the breeding period, birds seem to exhibit antipredatory behaviours when exposed to predator chemical cues inside the nest-boxes (Amo et al. 2008) or close to them (Amo et al. 2017). Predation is prey density dependent, i.e. predation efficiency increases with increasing prey density, and consequently predators develop a searching image and start to concentrate on more abundant prey. During the breeding period, bird density is higher than in winter (e.g. in Northeast Spain population, nest-box occupancy is 67% during the breeding period and only 22% during the winter) and predators may also obtain greater benefits when finding an occupied nest-box in spring than in winter. However, at least in Spanish populations, we have observed roosting birds killed by mammal predators inside the nest-boxes (L. Amo, personal observation, [44]). Therefore, predation pressure may be sufficiently important during the winter period to make birds detect the predator chemical cues. Previous evidence supports this hypothesis because wild birds avoid roosting in nest-boxes containing cues from predators such as fur or signs of predation such as bird feathers [19]. Also, in captivity, they avoid roosting in nest-boxes containing predator scent [20]. Therefore, differences in prey density and a potential decrease in the risk of predation in winter may not be responsible for the lack of an avoidance response observed in our studies in the wild.

The different results obtained in captivity and in the wild may, however, be explained by differences in experimental designs. In the experiment performed in captivity [40], captive great tits were released in an unknown aviary one and a half hour before sunset to allow them to inspect the aviary and the nest-boxes before choosing one for spending the night. Inside the aviary there were two nest-boxes, one control and one experimental. The experimental nest-box had the odour of a mustelid predator or a strong new odour without biological significance (cologne), the control nest-box contained no odour. When one of the cavities contained the odour of a predator, birds avoided the use of either of the two offered nest-boxes, and a greater number of birds slept outside the next boxes. However, there was no avoidance of nest-boxes when one of them contained a control odour. In contrast, in the experiment under natural conditions, scents were located in the nest-boxes during the morning, so birds had more time to inspect the area surrounding the nest-boxes to try to visually ascertain the presence of the predator.

Predator chemical cues may offer a first indication of predator presence, as scents may reveal the presence of predators [21]. However, these cues may remain in the area once the predator has gone. Therefore, by using only predator chemical cues, prey can overestimate the risk of predation if the predator is no longer present or is not willing to perform an attack [21, 39]. In contrast, other cues (e.g., visual) may provide prey with more current information about predator motivation for hunting and overall threat [39]. Despite that birds respond similarly to predator chemical cues alone than to visual cues alone [26], birds probably assess actual predation risk by integrating information from all available cue types, i.e. they detect predator cues but they probably confirm predator presence by trying to visually detect the predator. Therefore, if they did not find other cues signalling predator presence, they opted to use the nest-box despite it contained predator chemical cues. The lack of other cues signalling predator presence may explain that in another study, a long term avoidance response to predator scented nest-boxes has not been observed [32]. In contrast, in other studies using visual and chemical cues of predators, an avoidance response was found in the use of nest-boxes for roosting [19] or breeding [20].

The use of cavities for roosting in winter may be critical for small birds inhabiting temperate areas [45] because the energy saving thanks to the use of cavities to spend the night can influence survival [46, 47]. The energy saving due to the use of cavities compared to roosting in the canopy increases when the cavity contains nest material. In a study with tree sparrows (*Passer montanus*), roosting in empty nest-boxes can represent an energy saving of 18%, increasing to 36% in nest-boxes containing a nest [48]. Our results show that birds did not chose nest-boxes containing a nest. Previous studies have demonstrated that an old nest may contain ectoparasites, influencing bird preferences when selecting nest-boxes for roosting [49]. Therefore, our results suggest that the benefits in terms of energy saving may not overcome both the parasitism and the predation risks costs, as it has been found in other mainland populations in Europe [50, 51], for example in Corsica, where blue tits do not roost in nest-boxes [52]. In this area with evergreen forests, roosting in the dense foliage may be less energetically costly than roosting in deciduous forests that prevail in mainland [52]. Thus, differences in the balance between costs and benefits of roosting in cavities may have exerted different selection pressures that have led to population differences in the use of nest-boxes during the night [52].

In populations that use nest-boxes for roosting, previous evidence suggests that nest-boxes are the preferred roosting sites, because dominant males are found at higher proportions than subdominant, juvenile or female birds [53,54]. Furthermore, dominant species roost in nest-boxes at higher proportions than subdominant species [55]. For example, although blue tits prefer to roost in nest-boxes with big entrance holes, when great tits are present blue tits use small-holed nest-boxes [55]. In our study areas, the low occupancy rate of nest-boxes in the three populations (15–31%) suggests that nest-boxes are not a limited resource for roosting birds. Furthermore, treatments were balanced so a predator-scented nest-box was surrounded by a control-scented and a control-unscented nest-box. Nest-boxes were separated up to 40 m. Results of a previous study showed that great tits use nest-boxes for roosting within a territory of around 120 m in diameter [54]. So birds may not need to leave their territory to search for another nest-box where to roost. However, the thermal insulation properties of the other nest-boxes present in a territory may differ, for example due to exposition to the wind. What is more, previous evidence suggests that birds that usually roost in a particular nest-box, use it for breeding the following breeding season [19]. Therefore, the need to maintain a nest-box for the breeding season may also explain that birds did not search for another nest-box when they only found predator chemical cues inside their nest-box.

In conclusion, our results suggest that despite that birds are able to detect the predator scent and use it to assess the risk of predation inside cavities [25, 40], and they avoid roosting in nest-boxes containing predator chemical cues in captivity [40], in natural conditions, territory maintenance or thermoregulatory benefits of roosting in nest-boxes may overcome the perceived risk of predation when only predator chemical cues are present. Further research is needed to assess whether this lack of avoidance of roosting in predator scented nest-boxes is maintained when thermoregulatory costs of sleeping outside nest-boxes are lower.

## Supporting information

S1 Data Data of the experiments. “Forest” indicates the forest where the experiment was performed, “Treatment” (odourless control, odorous control and predator scent) indicates the scent assigned to each nest-box. “Order” indicates the order of scent application. “Nest” indicates whether the nest-box contained a nest. “Initial” indicates whether the nest-box was previously used, and “Use” indicates whether the nest-box was used for roosting after adding the treatment.
(XLSX)
